# Elevated Myoglobin in Patients With Primary Aldosteronism: A Cross-Sectional Study

**DOI:** 10.3389/fendo.2022.799174

**Published:** 2022-02-21

**Authors:** Bing Kang, Chuan Peng, Kanran Wang, Ying Song, Yi Yang, Linqiang Ma, Mei Mei, Jinbo Hu, Shumin Yang, Fei-Fei Wu, Qifu Li

**Affiliations:** ^1^ Department of Endocrinology, The First Affiliated Hospital of Chongqing Medical University, Chongqing, China; ^2^ Department of Clinical Nutrition, The First Affiliated Hospital of Chongqing Medical University, Chongqing, China; ^3^ The Chongqing Key Laboratory of Translational Medicine in Major Metabolic Diseases, The First Affiliated Hospital of Chongqing Medical University, Chongqing, China; ^4^ Department of Endocrinology, Affiliated Heping Hospital, Changzhi Medical College, Changzhi, China

**Keywords:** primary aldosteronism, markers, myoglobin, high sensitivity troponin T, creatine kinase isoenzyme MB

## Abstract

**Objectives:**

Primary aldosteronism (PA) is characterized by the autonomous excessive production of aldosterone in the adrenal cortex. Aldosterone is associated with damages to heart muscle and skeletal muscle. The purpose of this study was to evaluate serum levels of muscle injury markers and their associated factors in patients with primary aldosteronism.

**Methods:**

We retrospectively enrolled subjects with PA and essential hypertension (EH) who had completed testing for serum high sensitivity troponin T (hs-TnT), creatine kinase isoenzyme MB (CK-MB) and myoglobin from the database of the Chongqing Primary Aldosteronism Study (CONPASS). Univariate and multivariate linear regression analyses were performed to analyze the influencing factors of myocardial injury markers.

**Results:**

In total, 278 patients with PA and 445 patients with EH were enrolled in this study. Compared with EH patients, serum concentrations of hs-TnT [7.0 (4.0–12.0) *vs.* 6.0 (3.0–11.0) ng/L; *p*=0.005] and myoglobin [24.2 (21.0–38.1) *vs.* 21.8 (21.0–31.9) μg/L; *p*=0.023] were significantly higher among PA patients, while no significant difference of CK-MB was found between two groups [1.4 (1.0–2.0) *vs.* 1.3 (0.9–1.9) μg/L; *p*=0.154]. Univariate linear regression analysis showed that myoglobin was negatively correlated with serum potassium (β=-0.31; *p*<0.01) and positively correlated with plasma aldosterone concentration (β=0.40; *p*<0.01) in the PA group, while no significant correlation was found between hs-TnT and biochemical parameters. After adjusting for multiple confounders, myoglobin was negatively correlated with serum potassium (β=-0.15; *p*<0.05) and positively correlated with plasma aldosterone concentration (β=0.34; *p*<0.01) in the PA group.

**Conclusions:**

The serum level of myoglobin was significantly increased in PA patients, and myoglobin was independently correlated with plasma aldosterone concentration.

## Introduction

Primary aldosteronism (PA) is characterized by the autonomous excessive production of aldosterone in the adrenal cortex, resulting in sodium retention, potassium excretion, increased blood volume, and a suppressed renin-angiotensin system (1). The typical clinical manifestations of PA are hypertension with or without hypokalemia (2–4). A recent study has indicated that the prevalence of PA among hypertension is 5-10% (5). Patients with PA have a higher risk of cardiovascular events, kidney damage and all-cause mortality than those with essential hypertension (EH) who are matched for age, sex, and blood pressure (6). Excessive aldosterone concentrations promote the onset and progression of cardiovascular diseases through various mechanisms, such as chronic vascular fluid retention, endothelial dysfunction, target organ inflammation and fibrosis (7).

Cardiac troponin T (TnT), creatine kinase isoenzyme MB (CK-MB) and myoglobin are widely used as cardiac injury markers (8, 9). Previous studies have shown that CK-MB and myoglobin are mainly distributed in the myocardium and skeletal muscle, and increase after damage to the myocardium or skeletal muscle cells, and hs-TnT is not only significantly elevated in acute coronary syndrome, but also reflects chronic myocardial injury or subclinical myocardial injury of unknown causes (10). hs-TnT, CK-MB and myoglobin are associated with chronic diseases such as diabetes and end-stage renal disease (11–15). Previous study showed that aldosterone is associated with damages to heart and skeletal muscle cells (16–19), while very few studies have evaluated serum levels of muscle injury markers in PA patients.

The present study aimed to compare serum levels of hs-TnT, CK-MB, myoglobin between patients with PA and EH, and analyze whether these markers correlated with clinical parameters such as aldosterone, which may facilitate seeking for biomarkers of hyperaldosteronemia related myocardium injury.

## Subjects and Methods

### Study Population

We retrospectively enrolled patients diagnosed with PA and EH patients from the database of the Chongqing Primary Aldosteronism Study (CONPASS) (20–23) at the First Affiliated Hospital of Chongqing Medical University in China from November 2013 to January 2020. The inclusion criteria were as follows: patients aged 18 to 75 years; patients who had completed testing for serum hs-TnT, CK-MB and myoglobin. The exclusion criteria were as follows: 1) other known causes of secondary hypertension; 2) acute cardiovascular events within 3 months, including myocardial infarction and angina pectoris; 3) chronic kidney disease, defined as an estimated glomerular filtration rate (eGFR)<60 mL/min/1.73 m^2^, which was calculated using the Chronic Kidney Disease Epidemiology Collaboration (CKD-EPI) equation (24); 4) acute or chronic heart failure (according to the New York Heart Association functional classification) (25, 26); 5) chronic obstructive pulmonary disease.

Clinical information, including medical history was collected through physician interviews. All subjects had undergone a physical examination including measurement of the height, weight and blood pressure. Hypokalemia was defined as a serum potassium level <3.5 mmol/L. Antihypertensive medications are showed as defined daily dose (DDD), which is the assumed average maintenance dose per day (27). Patients with cardiovascular diseases (CVD) 3 months before enrollment were considered with preexisting CVD. Target organ damage is defined as the presence of left ventricular hypertrophy at echocardiography and/or microalbuminuria (28). The local ethics committee approved the protocol, and the informed consent was obtained from all the participants.

### Screening and Confirmatory Tests for PA

Before screening and confirmatory tests, antihypertensive medication was withdrawn or changed according to the Endocrine Society’s clinical practice guideline (1). Only non-dihydropyridine calcium channel blockers, terazosin, and doxazosin were allowed for uncontrolled hypertension. For screening, blood samples for plasma renin concentration (PRC) and the plasma aldosterone concentration were collected in the morning after the subjects were out of bed and maintained an upright position for at least 2 hours. Aldosterone-to-renin ratio (ARR) was calculated as plasma aldosterone concentration divided by PRC. ARR>20 pg·mL^-1^/μIU·mL^-1^ (27 pmol·L^-1^/μIU·mL^-1^) was considered a positive screening test (29). Patients who tested positive for ARR or negative but were strongly suspected of having PA were subjected to the confirmatory tests, while those who tested negative were not considered PA.

Confirmatory tests were performed as described in the current guidelines (1, 30), and PA was considered if any of the following criteria were met: 1) plasma aldosterone concentration > 110 pg/mL (305 pmol/L) after the captopril challenge test (CCT) (22); 2) plasma aldosterone concentration >80 pg/mL (221 pmol/L) after the infusion of 2 L normal saline in the recumbent position (22) or >85 pg/mL (235 pmol/L) in the seated position (21); 3) plasma aldosterone concentration > 60 pg/mL (166 pmol/L) on the fourth day of the fludrocortisone suppression test (FST).

### Biochemical Measurements

Blood samples were collected to measure plasma aldosterone concentration, PRC, hs-TnT, CK-MB and myoglobin. Blood electrolytes were measured using an indirect ion selective electrode method and a Hitachi 7600-020 machine. hs-TnT, CK-MB and myoglobin were measured at 37°C using the electrochemiluminescence method, the instrument used was Roche E602, and the manufacturer’s kit was from Roche. Plasma aldosterone concentration and PRC were measured using the automatic chemiluminescence immunoassay (DiaSorin, Liaison, Italy), the detection range of plasma aldosterone concentration was 2.2~100 ng/dL, and the differences were 2.4%~4.8% and 4.4%~6.7% for inter-batch and intra-batch samples, respectively. The detection range of PRC was 0.13~0.53 mU/L, and the intrabatch and interbatch differences were 1.2%~3.7% and 2.9%~12.8%, respectively.

### Statistical Analysis

SPSS 22.0 analysis software was used for statistical analysis. The measurement data were tested for normality and homogeneity of variance. Normally distributed variables were expressed as means ± standard deviation, nonnormally distributed variables as medians (interquartile range), and categorical variables as absolute values and percentages. Independent sample t test was used to compare the normally distributed data between the two groups, and χ2 test was used to compare the counting data. Univariate linear regression was used to analyze the correlation between the three markers and the clinical/biochemical parameters of the enrolled subjects. *P*<0.05 indicated a statistically significant difference.

## Results

### Characteristics of the Study Participants

The demographic, clinical and biological characteristics of the study population are summarized in **Table 1**. In total, 278 patients with PA and 445 patients with EH were enrolled in the present study. No significant difference was found in age, sex, BMI, diabetes, preexisting CVD, hs-CRP, or CK-MB between the PA and EH groups (*p*>0.05). Compared with EH patients, the SBP (153 ± 20 *vs.* 146 ± 23 mmHg; *p*<0.001), DBP (92 ± 14 *vs.* 89 ± 16 mmHg; *p*=0.01), DDD [1.5(1–2) *vs.* 1(0-2); *p*<0.001], duration of hypertension [7(2–13) *vs.* 3(0–8) years; *p*<0.001], serum creatinine (Scr) [71(58–89) *vs.* 68(57–83) µmol/L; *p*=0.045], history of hypokalemia (74.1% *vs.* 31.2%; *p*<0.001), plasma aldosterone concentration [270.0(192.0–401.0) *vs.* 116.0(72.3–171.0) pg/mL; *p*<0.001], ARR [100.3(33.2–319.2) *vs.* 7.2(3.2–21.7) pg·mL^1^/μIU·mL^-1^; *p*<0.001], hs-TnT [7.0 (4.0–12.0) *vs.* 6.0(3.0–11.0) ng/L; *p*=0.005] and myoglobin [24.2(21.0–38.1) *vs.* 21.8(21.0–31.9) μg/L; *p*=0.023] were significantly higher in patients with PA. The proportion of target organ damage (31.7% *vs.* 15.1%; *p*<0.001) was higher in PA patients. The serum potassium concentration [3.3(2.9–3.8) *vs.* 3.9(3.6–4.2) μg/L; *p*<0.001] and PRC [2.5(0.9–7.0) *vs.* 12.8(5.1–32.2) μIU/mL; *p*<0.001] were lower in PA patients.

**Table 1:** Demographic and clinical characteristics of the patient cohorts.

Characteristics	PA (n=278)	EH (n=445)	P-value
Age (years)	51 ± 12	51 ± 14	0.497
Sex (women, %)	58.3%	55.1%	0.441
BMI (kg/m^2^)	24.8 (22.3-27.0)	24.7 (22.5-27.3)	0.435
SBP (mmHg)	153 ± 20	146 ± 23	<0.001
DBP (mmHg)	92 ± 14	89 ± 16	0.01
Antihypertensive medication (DDD)	1.5 (1.0-2.0)	1.0 (0-2.0)	<0.001
Duration of hypertension (years)	7 (2-13)	3 (0-8)	<0.001
Diabetes, n (%)	66 (23.7%)	124 (27.9%)	0.226
Preexisting CVD, n (%)	39 (14.0%)	48 (10.8%)	0.198
Target organ damage, n (%)	88 (31.7%)	67 (15.1%)	<0.001
History of hypokalemia, n (%)	206 (74.1%)	139 (31.2%)	<0.001
Scr (umol/L)	71 (58-89)	68 (57-83)	0.045
Serum K^+^ (mmol/L)	3.3 (2.9-3.8)	3.9 (3.6-4.2)	<0.001
hs-CRP (mg/L)	1.1 (0.5-2.3)	1.0 (0.4-2.5)	0.616
Plasma aldosterone concentration (pg/mL)	270.0 (192.0-401.0)	116.0 (72.3-171.0)	<0.001
PRC (μIU/mL)	2.5 (0.9-7.0)	12.8 (5.1-32.2)	<0.001
ARR (pg·mL^-1^/μIU·mL^-1^)	100.3 (33.2-319.2)	7.2 (3.2-21.7)	<0.001
hs-cTnT (ng/L)	7.0 (4.0-12.0)	6.0 (3.0-11.0)	0.005
CK-MB (μg/L)	1.4 (1.0-2.0)	1.3 (0.9-1.9)	0.154
Myoglobin (μg/L)	24.2 (21.0-38.1)	21.8 (21.0-31.9)	0.023

Data were presented as mean ± SD, %, or median (interquartile range). BMI, body index mass; SBP, systolic blood pressure; DBP, diastolic blood pressure; DDD, defined daily dose; CVD, cardiovascular diseases; Scr, serum creatinine; hs-CRP, high sensitivity C-reactive protein; PRC, plasma renin concentration; ARR, plasma aldosterone/renin ratio; hs-cTnT, high sensitivity troponin T; CK-MB, creatine kinase isoenzyme MB.

### hs-TnT, CK-MB and Myoglobin With Correlative Factors

Univariate linear regression analysis showed that myoglobin was negatively correlated with serum potassium (β=-0.31, *p*<0.01), and positively correlated with plasma aldosterone concentration (β=0.40; *p*<0.01) and ARR (β=0.37, *p*<0.01) in the PA group, while no significant correlation was found with age, SBP, DBP, DDD, duration of hypertension, the proportion of diabetes, preexisting CVD, target organ damage or serum creatinine concentration(*p*>0.05). None of the three markers (hs-TnT, CK-MB and myoglobin) were significantly associated with SBP, DBP, duration of hypertension, serum creatinine concentration, serum potassium concentration, plasma aldosterone concentration or ARR in the EH group (*p*>0.05) (**Table 2**).

**Table 2:** Univariate linear regression between myocardial injury markers and clinical characteristics in the study participants.

Characteristics	PA (n=278)			Non-PA (n=445)			All the crowd (n=723)		
	hs-cTnT	CK-MB	Myoglobin	hs-cTnT	CK-MB	Myoglobin	hs-cTnT	CK-MB	Myoglobin
	β	β	β	β	β	β	β	β	β
Age (years)	-0.04	-0.04	-0.03	0.03	-0.07	-0.07	0.02	-0.04	-0.04
BMI (kg/m^2^)	-0.01	-0.15^*^	-0.12	0.03	-0.02	-0.01	0.02	-0.09^*^	-0.07
SBP (mmHg)	0.01	0.03	0.09	-0.05	-0.02	0.03	-0.04	0.03	0.07
DBP (mmHg)	0.01	0.05	0.04	0.01	-0.03	0.05	0.01	0.03	0.05
Antihypertensive medication (DDD)	-0.10	-0.04	-0.02	-0.01	-0.07	-0.02	-0.03	-0.03	0.01
Duration of hypertension (years)	-0.05	0.03	0.01	0.01	-0.04	-0.05	-0.01	0.03	0.01
Diabetes	-0.03	-0.07	-0.09	0.09	-0.02	-0.05	0.07	-0.05	-0.07
Preexisting CVD	-0.02	0.09	0.07	0.12^*^	-0.01	-0.03	0.08^*^	0.06	0.03
Target organ damage	-0.04	-0.02	0.05	-0.03	-0.01	0.01	-0.03	0.01	0.05
Scr (umol/L)	-0.02	-0.01	0.01	0.06	0.04	0.16^**^	0.04	0.02	0.07
Serum K^+^ (mmol/L)	-0.09	-0.28^**^	-0.31^**^	0.07	-0.07	-0.07	0.04	-0.23^**^	-0.24^**^
hs-CRP (mg/L)	-0.02	0.00	0.12	-0.03	0.14^**^	0.08	-0.02	0.04	0.09^*^
Plasma aldosterone concentration(pg/mL)	0.06	0.38^**^	0.40^**^	-0.04	0.04	0.04	0.03	0.30^**^	0.31^**^
PRC (μIU/mL)	-0.03	-0.07	-0.07	-0.02	0.20^**^	0.09	-0.01	0.06	0.01
ARR (pg·mL^-1^/μIU·mL^-1^)	0.06	0.41^**^	0.37^**^	-0.01	0.01	0.01	-0.01	0.35^**^	0.32^**^

^**^P < 0.01, ^*^P < 0.05.

After adjusting for age, sex, BMI, SBP, DBP, DDD, duration of hypertension, the proportion of diabetes, preexisting CVD, and target organ damage, multivariate linear regression analysis showed that myoglobin was negatively correlated with serum potassium (β=-0.15; *p*<0.05), and positively correlated with plasma aldosterone concentration (β=0.34; *p*<0.01), while no significant correlation was observed between hs-TnT and serum potassium or plasma aldosterone concentration in the PA group(*p*>0.05). No correlation was found between the three markers (hs-TnT, CK-MB and myoglobin) and serum potassium or plasma aldosterone concentration in the EH group (*p*>0.05) (**Table 3**).

**Table 3:** Multivariate linear regression between myocardial injury markers and biochemical characteristics in the study participants.

Characteristics	PA (n=278)			Non-PA (n=445)			All the crowd (n=723)		
	hs-cTnT	CK-MB	Myoglobin	hs-cTnT	CK-MB	Myoglobin	hs-cTnT	CK-MB	Myoglobin
	β	β	β	β	β	β	β	β	β
Serum K^+^ (mmol/L) ^#^	-0.09	-0.13^*^	-0.15^*^	0.04	-0.09	-0.05	0.01	-0.11^**^	-0.11^**^
Plasma aldosterone concentration (pg/mL) ^##^	0.02	0.32^**^	0.34^**^	-0.03	0.05	0.04	-0.01	0.28^**^	0.26^**^

^#^Adjusted for age, sex, BMI, SBP, DBP, DDD, duration of hypertension, diabetes, preexisting CVD, target organ damage, and plasma aldosterone concentration.

^##^Adjusted for age, sex, BMI, SBP, DBP, DDD, duration of hypertension, diabetes, preexisting CVD, target organ damage, and serum K^+^.

^**^P < 0.01, ^*^P < 0.05.

## Discussion

Previous study showed that aldosterone is associated with damages to heart and skeletal muscle cells (16–19), while very few studies have evaluated serum levels of muscle injury markers in PA patients. For the first time, we compared serum levels of hs-TnT, CK-MB and myoglobin between patients with PA and EH. We found that hs-TnT and myoglobin were higher in PA patients than that in EH patients. Univariate linear regression analysis showed that myoglobin is negatively associated with serum potassium and positively associated with plasma aldosterone concentration in PA patients. The relationship existed after adjusting for some potential confounders, especially the serum potassium, blood pressure, DDD, duration of hypertension, the proportion of diabetes, preexisting CVD, and target organ damage and age.

hs-TnT is specific and sensitive biomarkers of myocardial injury. It is the preferred serologic tests for the evaluation of patients with suspected acute myocardial infarction (31–33). In addition to acute myocardial injury, previous studies have found that serum hs-TnT is increased in many chronic diseases, such as chronic heart failure, pulmonary hypertension, stable coronary heart disease, and chronic kidney disease (34, 35). Our study found that hs-TnT increased in PA patients when compared with subjects with EH. However, no significant correlation was found between serum hs-TnT and plasma aldosterone concentration or other clinical parameters, suggesting that hs-TnT elevation in PA patients may be resulted from other underlying factors. In addition to hs-TnT, high sensitivity troponin I (hs-TnI) has also been reported to be associated with nonfatal myocardial infarction (36), whether hs-TnI is superior to hs-TnT for indicating aldosterone induced myocardial injury requires more studies.

Creatine kinase is an important energy-regulating enzyme in muscle tissues that catalysis creatine-generated creatine phosphate and ADP with the energy provided by ATP. Creatine kinase is a dimer comprising two subunits, M and B, and CK-MB mainly exists in the myocardium and skeletal muscle (37). CK-MB are elevated when muscle cells are damaged, which included acute myocardial infarction, myocarditis and myositis (38). However, we did not find higher CK-MB in PA patients than EH in our study. Although there is a correlation between CK-MB and plasma aldosterone concentration, it might have little clinical significance.

Interestingly, our study found myoglobin was increased in PA patients when compared with subjects with EH. In fact, patients with disease which might obviously influence the three markers were excluded from our study. The increase of myoglobin found here in PA patients was not as high as in acute myocardial injury or rhabdomyolysis, and it did not reflect acute muscle injury. Myoglobin is a cytoplasmic hemoprotein that is synthesized in cardiomyocytes and skeletal muscle cells. It is an oxygen storage protein, capable of releasing oxygen during periods of hypoxia or anoxia (39, 40). The serum levels of myoglobin increase in acute and chronic muscle injures and decrease with age as the muscle mass becomes less in older people (41, 42). In our study, when age, BMI, blood pressure and serum potassium were adjusted, myoglobin is still positively correlated with plasma aldosterone concentration in PA patients. This is a retrospective study, and it is not clear which of the high aldosterone level and the increased myoglobin level observed in the study came first. It has been reported that aldosterone can directly damage skeletal and cardiac muscle cells (16, 18, 19), while aldosterone receptor antagonists can improve the injury of muscle cells (16, 43–46). Although we found that myoglobin was independently associated with plasma aldosterone concentration in PA patients, whether the level of myoglobin might change after mineralocorticoid receptor antagonists (MRA) treatment was not clear. Aldosterone receptors are widely expressed in tissues and cells throughout the body, including cardiac and skeletal muscle cells. There is a possibility that excessive production of aldosterone in the adrenal cortex may directly or indirectly caused myoglobin release in muscle cells. However, the hypothesis needs further study to verify.

Several limitations in this study should be mentioned. First, this is a retrospective study and the comparison of muscle injury markers in EH and PA cohorts might be affected by potential differences in the underlying phenotypes of the two cohorts. However, clinical characteristics including age, sex and BMI were similar between the two cohorts. Second, this is a cross-sectional study, although a positive association was found between plasma aldosterone concentration and myoglobin, causal relationship between them could not be answered by this study. Third, myoglobin exists not only in in cardiac muscles but also in skeletal muscles and which one is the source of the elevated myoglobin in PA patients was not clear. In addition, other indicators of muscle damage and potential confounding factors such as total creatine kinase, magnesium and calcium ions were not detected in this study.

## Conclusion

The serum level of myoglobin was significantly increased in PA patients, and myoglobin was independently correlated with plasma aldosterone concentration, which might be a reflection of chronic muscle injury in PA patient.

## Data Availability Statement

The original contributions presented in the study are included in the article/supplementary material. Further inquiries can be directed to the corresponding authors.

## Ethics Statement

The studies involving human participants were reviewed and approved by Institutional Review Board of the First Affiliated Hospital of Chongqing Medical University. The patients/participants provided their written informed consent to participate in this study.

## Author Contributions

QL, F-FW, JH, and SY contributed to conception and design of the study. JH and SY supervised the study. YS, YY, MM, and LM organized the database. BK, KW, and CP performed the statistical analysis. BK and CP wrote the first draft of the manuscript. KW and YY wrote the sections of the manuscript. All authors contributed to article and approved the submitted version.

## Funding

This study is funded by the National Natural Science Foundation of China (81670785, 81800701, 81870567, 81800731 and 81970720), and the National Key Research & Development Plan, major project of precision medicine research (2017YFC0909600) and Chongqing Science and Technology Committee Innovation Project (Technology Development and Application of Precision Medicine, cstc2016shms-ztzx1003) and Joint Medical Research Project of Chongqing Science and Technology Commission & Chongqing Health and Family Planning Commission (Youth Project, 2018QNXM001) and Outstanding Talents of the First Affiliated Hospital of Chongqing Medical University 2019 (2019-4-22) and Chongqing Outstanding Youth Funds(cstc2019jcyjjq0006).

## Conflict of Interest

The authors declare that the research was conducted in the absence of any commercial or financial relationships that could be construed as a potential conflict of interest.

## Publisher’s Note

All claims expressed in this article are solely those of the authors and do not necessarily represent those of their affiliated organizations, or those of the publisher, the editors and the reviewers. Any product that may be evaluated in this article, or claim that may be made by its manufacturer, is not guaranteed or endorsed by the publisher.

## References

[1] Funder JW Carey RM Mantero F Murad MH Reincke M Shibata H. The Management of Primary Aldosteronism: Case Detection, Diagnosis, and Treatment: An Endocrine Society Clinical Practice Guideline. J Clin Endocrinol Metab (2016) 101(5):1889–916. doi: 10.1210/jc.2015-4061 26934393

[2] Calhoun DA Nishizaka MK Zaman MA Thakkar RB Weissmann P. Hyperaldosteronism Among With Resistant Black and White Subjects Hypertension. Hypertension (2002) 40(6):892–6. doi: 10.1161/01.Hyp.0000040261.30455.B6 12468575

[3] Kayser SC Dekkers T Groenewoud HJ van der Wilt GJ Bakx JC van der Wel MC. Study Heterogeneity and Estimation of Prevalence of Primary Aldosteronism: A Systematic Review and Meta-Regression Analysis. J Clin Endocr Metab (2016) 101(7):2826–35. doi: 10.1210/jc.2016-1472 27172433

[4] Monticone S Burrello J Tizzani D Bertello C Viola A Buffolo F. Prevalence and Clinical Manifestations of Primary Aldosteronism Encountered in Primary Care Practice. J Am Coll Cardiol (2017) 69(14):1811–20. doi: 10.1016/j.jacc.2017.01.052 28385310

[5] Xu ZX Yang J Hu JB Song Y He WW Luo T. Primary Aldosteronism in Patients in China With Recently Detected Hypertension. J Am Coll Cardiol (2020) 75(16):1913–22. doi: 10.1016/j.jacc.2020.02.052 32327102

[6] Monticone S D’Ascenzo F Moretti C Williams TA Veglio F Gaita F. Cardiovascular Events and Target Organ Damage in Primary Aldosteronism Compared With Essential Hypertension: A Systematic Review and Meta-Analysis. Lancet Diabetes Endocrinol (2018) 6(1):41–50. doi: 10.1016/s2213-8587(17)30319-4 29129575

[7] Gaddam KK Pimenta E Husain S Calhoun DA. Aldosterone and Cardiovascular Disease. Curr Problems Cardiol (2009) 34(2):51–84. doi: 10.1016/j.cpcardiol.2008.10.002 19135616

[8] Thygesen K Alpert JS White HD. Universal Definition of Myocardial Infarction. J Am Coll Cardiol (2007) 50(22):2173–95. doi: 10.1016/j.jacc.2007.09.011 18036459

[9] Thygesen K Alpert JS Jaffe AS Simoons ML Chaitman BR White HD. Third Universal Definition of Myocardial Infarction. Eur Heart J (2012) 33(20):2551–67. doi: 10.1093/eurheartj/ehs184 22922414

[10] Hammarsten O Mair J Möckel M Lindahl B Jaffe AS. Possible Mechanisms Behind Cardiac Troponin Elevations. Biomark: Biochem Indic Exposure Response Susceptibility To Chemicals (2018) 23(8):725–34. doi: 10.1080/1354750x.2018.1490969 29976112

[11] Zheng J Ye P Luo L Xiao W Xu R Wu H. Association Between Blood Glucose Levels and High-Sensitivity Cardiac Troponin T in an Overt Cardiovascular Disease-Free Community-Based Study. Diabetes Res Clin Pract (2012) 97(1):139–45. doi: 10.1016/j.diabres.2012.04.021 22579531

[12] Selvin E Lazo M Chen Y Shen L Rubin J McEvoy JW. Diabetes Mellitus, Prediabetes, and Incidence of Subclinical Myocardial Damage. Circulation (2014) 130(16):1374–82. doi: 10.1161/circulationaha.114.010815 PMC4198442 25149362

[13] Jaffe AS Lindahl B Giannitsis E Mueller C Cullen L Hammarsten O. ESC Study Group on Cardiac Biomarkers of the Association for Acute CardioVascular Care: A Fond Farewell at the Retirement of CKMB. Eur Heart J (2021) 42(23):2260–2264. doi: 10.1093/eurheartj/ehaa1079 33486520

[14] Iliou MC Fumeron C Benoit MO Tuppin P Calonge VM Moatti N. Prognostic Value of Cardiac Markers in ESRD: Chronic Hemodialysis and New Cardiac Markers Evaluation (CHANCE) Study. Am J Kidney Dis: Off J Natl Kidney Foundation (2003) 42(3):513–23. doi: 10.1016/s0272-6386(03)00746-7 12955679

[15] Mutluay R Konca C Erten Y Paşaoğlu H Değer SM Ağirgün C. Predictive Markers of Asymptomatic Atherosclerosis in End-Stage Renal Disease Patients. Renal Failure (2010) 32(4):448–54. doi: 10.3109/08860221003658258 20446782

[16] Burniston JG Saini A Tan LB Goldspink DF. Aldosterone Induces Myocyte Apoptosis in the Heart and Skeletal Muscles of Rats *In Vivo* . J Mol Cell Cardiol (2005) 39(2):395–9. doi: 10.1016/j.yjmcc.2005.04.001 15907929

[17] Lastra G Whaley-Connell A Manrique C Habibi J Gutweiler AA Appesh L. Low-Dose Spironolactone Reduces Reactive Oxygen Species Generation and Improves Insulin-Stimulated Glucose Transport in Skeletal Muscle in the TG(mRen2)27 Rat. Am J Physiol Endocrinol Metab (2008) 295(1):E110–6. doi: 10.1152/ajpendo.00258.2007 PMC2493595 18445755

[18] Selvaraj J Muthusamy T Srinivasan C Balasubramanian K. Impact of Excess Aldosterone on Glucose Homeostasis in Adult Male Rat. Clinica Chimica Acta; Int J Clin Chem (2009) 407(1-2):51–7. doi: 10.1016/j.cca.2009.06.030 19567248

[19] Selvaraj J Sathish S Mayilvanan C Balasubramanian K. Excess Aldosterone-Induced Changes in Insulin Signaling Molecules and Glucose Oxidation in Gastrocnemius Muscle of Adult Male Rat. Mol Cell Biochem (2013) 372(1-2):113–26. doi: 10.1007/s11010-012-1452-2 23007523

[20] Shu X Mei M Ma L Wang Z Yang S Hu J. Postmenopausal Osteoporosis Is Associated With Elevated Aldosterone/Renin Ratio. J Hum Hypertension (2018) 32(7):524–30. doi: 10.1038/s41371-018-0069-7 29789689

[21] Wang K Hu J Yang J Song Y Fuller PJ Hashimura H. Development and Validation of Criteria for Sparing Confirmatory Tests in Diagnosing Primary Aldosteronism. J Clin Endocrinol Metab (2020) 105(7):e2449–56. doi: 10.1210/clinem/dgaa282 32449927

[22] Song Y Yang S He W Hu J Cheng Q Wang Y. Confirmatory Tests for the Diagnosis of Primary Aldosteronism: A Prospective Diagnostic Accuracy Study. Hypertension (Dallas Tex: 1979) (2018) 71(1):118–24. doi: 10.1161/hypertensionaha.117.10197 29158354

[23] Lin C Yang J Fuller PJ Jing H Song Y He W. A Combination of Captopril Challenge Test After Saline Infusion Test Improves Diagnostic Accuracy for Primary Aldosteronism. Clin Endocrinol (2020) 92(2):131–7. doi: 10.1111/cen.14134 31774187

[24] Levey AS Stevens LA Schmid CH Zhang YL Castro AF 3rd Feldman HI. A New Equation to Estimate Glomerular Filtration Rate. Ann Internal Med (2009) 150(9):604–12. doi: 10.7326/0003-4819-150-9-200905050-00006 PMC2763564 19414839

[25] Yancy CW Jessup M Bozkurt B Butler J Casey DE Jr Colvin MM. ACC/AHA/HFSA Focused Update of the 2013 ACCF/AHA Guideline for the Management of Heart Failure: A Report of the American College of Cardiology/American Heart Association Task Force on Clinical Practice Guidelines and the Heart Failure Society of America. J Cardiac Failure (2017) 23(8):628–51. doi: 10.1016/j.cardfail.2017.04.014 28461259

[26] Ponikowski P Voors AA Anker SD Bueno H Cleland JG Coats AJ. ESC Guidelines for the Diagnosis and Treatment of Acute and Chronic Heart Failure: The Task Force for the Diagnosis and Treatment of Acute and Chronic Heart Failure of the European Society of Cardiology (ESC). Developed With the Special Contribution of the Heart Failure Association (HFA) of the ESC. Eur J Heart Failure (2016) 18(8):891–975. doi: 10.1002/ejhf.592 27207191

[27] Williams TA Lenders JWM Mulatero P Burrello J Rottenkolber M Adolf C. Outcomes After Adrenalectomy for Unilateral Primary Aldosteronism: An International Consensus on Outcome Measures and Analysis of Remission Rates in an International Cohort. Lancet Diabetes Endocrinol (2017) 5(9):689–99. doi: 10.1016/s2213-8587(17)30135-3 PMC5572673 28576687

[28] Burrello J Amongero M Buffolo F Sconfienza E Forestiero V Burrello A. Development of a Prediction Score to Avoid Confirmatory Testing in Patients With Suspected Primary Aldosteronism. J Clin Endocrinol Metab (2021) 106(4):e1708–16. doi: 10.1210/clinem/dgaa974 33377974

[29] Ma L Song Y Mei M He W Hu J Cheng Q. Age-Related Cutoffs of Plasma Aldosterone/Renin Concentration for Primary Aldosteronism Screening. Int J Endocrinol (2018) 2018:8647026. doi: 10.1155/2018/8647026 30123268 PMC6079585

[30] Funder JW Carey RM Fardella C Gomez-Sanchez CE Mantero F Stowasser M. Case Detection, Diagnosis, and Treatment of Patients With Primary Aldosteronism: An Endocrine Society Clinical Practice Guideline. J Clin Endocrinol Metab (2008) 93(9):3266–81. doi: 10.1210/jc.2008-0104 18552288

[31] Amsterdam EA Wenger NK Brindis RG Casey DE Jr Ganiats TG Holmes DR Jr. AHA/ACC Guideline for the Management of Patients With Non-ST-Elevation Acute Coronary Syndromes: A Report of the American College of Cardiology/American Heart Association Task Force on Practice Guidelines. J Am Coll Cardiol (2014) 64(24):e139–228. doi: 10.1016/j.jacc.2014.09.017 25260718

[32] Roffi M Patrono C Collet JP Mueller C Valgimigli M Andreotti F. ESC Guidelines for the Management of Acute Coronary Syndromes in Patients Presenting Without Persistent ST-Segment Elevation: Task Force for the Management of Acute Coronary Syndromes in Patients Presenting Without Persistent ST-Segment Elevation of the European Society of Cardiology (ESC). Eur Heart J (2016) 37(3):267–315. doi: 10.1093/eurheartj/ehv320 26320110

[33] Thygesen K Alpert JS Jaffe AS Chaitman BR Bax JJ Morrow DA. Fourth Universal Definition of Myocardial Infarction (2018). Global Heart (2018) 13(4):305–38. doi: 10.1016/j.gheart.2018.08.004 30154043

[34] Carda R Aceña Á Pello A Cristóbal C Tarín N Huelmos A. The Prognostic Value of High-Sensitive Troponin I in Stable Coronary Artery Disease Depends on Age and Other Clinical Variables. Cardiology (2015) 132(1):1–8. doi: 10.1159/000381259 25997694

[35] Park KC Gaze DC Collinson PO Marber MS. Cardiac Troponins: From Myocardial Infarction to Chronic Disease. Cardiovasc Res (2017) 113(14):1708–18. doi: 10.1093/cvr/cvx183 PMC5852618 29016754

[36] Omland T Pfeffer MA Solomon SD de Lemos JA Røsjø H Šaltytė Benth J. Prognostic Value of Cardiac Troponin I Measured With a Highly Sensitive Assay in Patients With Stable Coronary Artery Disease. J Am Coll Cardiol (2013) 61(12):1240–9. doi: 10.1016/j.jacc.2012.12.026 23414791

[37] Xu C Zhang T Zhu B Cao Z. Diagnostic Role of Postmortem CK-MB in Cardiac Death: A Systematic Review and Meta-Analysis. Forensic Sci Med Pathol (2020) 16(2):287–94. doi: 10.1007/s12024-020-00232-5 32193705

[38] Singh G Baweja PS. Creatine Kinase-MB: The Journey to Obsolescence. Am J Clin Pathol (2014) 141(3):415–9. doi: 10.1309/ajcpbik3g4bwejko 24515770

[39] Zafar Gondal A Foris LA Richards JR. Serum Myoglobin. StatPearls. Treasure Island (FL: StatPearls Publishing Copyright © 2021, StatPearls Publishing LLC (2021). 29262167

[40] Ordway GA Garry DJ. Myoglobin: An Essential Hemoprotein in Striated Muscle. J Exp Biol (2004) 207(Pt 20):3441–6. doi: 10.1242/jeb.01172 15339940

[41] Beyer RE Fattore JE. The Influence of Age and Endurance Exercise on the Myoglobin Concentration of Skeletal Muscle of the Rat. J Gerontol (1984) 39(5):525–30. doi: 10.1093/geronj/39.5.525 6470441

[42] Garry DJ Mammen PP. Molecular Insights Into the Functional Role of Myoglobin. Adv Exp Med Biol (2007) 618:181–93. doi: 10.1007/978-0-387-75434-5_14 18269197

[43] Aagaard NK Andersen H Vilstrup H Clausen T Jakobsen J Dørup I. Muscle Strength, Na,K-Pumps, Magnesium and Potassium in Patients With Alcoholic Liver Cirrhosis – Relation to Spironolactone. J Internal Med (2002) 252(1):56–63. doi: 10.1046/j.1365-2796.2002.01008.x 12074739

[44] Farquharson CA Struthers AD. Spironolactone Increases Nitric Oxide Bioactivity, Improves Endothelial Vasodilator Dysfunction, and Suppresses Vascular Angiotensin I/angiotensin II Conversion in Patients With Chronic Heart Failure. Circulation (2000) 101(6):594–7. doi: 10.1161/01.cir.101.6.594 10673249

[45] Ramírez E Klett-Mingo M Ares-Carrasco S Picatoste B Ferrarini A Rupérez FJ. Eplerenone Attenuated Cardiac Steatosis, Apoptosis and Diastolic Dysfunction in Experimental Type-II Diabetes. Cardiovasc Diabetol (2013) 12:172. doi: 10.1186/1475-2840-12-172 24261558 PMC4222723

[46] Hung CS Chang YY Tsai CH Liao CW Peng SY Lee BC. Aldosterone Suppresses Cardiac Mitochondria. Trans Res: J Lab Clin Med (2021) 239:58–70. doi: 10.1016/j.trsl.2021.08.003 34411778

